# The Role of Geography in the Assessment of Quality: Evidence from the Medicare Advantage Program

**DOI:** 10.1371/journal.pone.0145656

**Published:** 2016-01-04

**Authors:** Rene Soria-Saucedo, Peng Xu, Jack Newsom, Howard Cabral, Lewis E. Kazis

**Affiliations:** 1 The Center for the Assessment of Pharmaceutical Practices (CAPP), Department of Health Policy and Management, Boston University School of Public Health, Boston, United States of America; 2 CenseoHealth, Dallas, United States of America; 3 Department of Biostatistics, Boston University School of Public Health, Boston, United States of America; National Institute of Health, ITALY

## Abstract

The Affordable Care Act set in motion a renewed emphasis on quality of care evaluation. However, the evaluation strategies of quality by the Centers for Medicare and Medicaid Services do not consider geography when comparisons are made among plans. Using an overall measure of a plan’s quality in the public sector—the Medicare Advantage (MA) star ratings—we explored the impact of geography in these ratings. We identified 2,872 U.S counties in 2010. The geographic factor predicted a larger fraction of the MA ratings’ compared to socio-demographic factors which explained less. Also, after the risk adjustments, almost half of the U.S. states changed their ranked position in the star ratings. Further, lower MA star ratings were identified in the Southeastern region. These findings suggest that the geographic component effect on the ratings is not trivial and should be considered in future adjustments of the metric, which may enhance the transparency, accountability, and importantly level the playing field more effectively when comparing quality across health plans.

## Introduction

Geographic variation has long been considered as an important case-mix variable in its relationship with patient and clinical outcomes [1]. A number of factors explain the importance of geographic variation in measuring health status. First, many systems of health care are organized on a geographical basis. Hence, distribution of health care resources are tailored to respond to local demands. Second, health care facilities such as hospitals and clinics are concentrated in specific locations, turning geography into a predictor of health utilization and outcomes. Third, there’s substantial evidence that “area effects” are drivers of health inequality, after controlling for social and economic factors. These “area effects” represent relationships between area characteristics and individual behavior which can’t be explained by individual attributes alone [2]. Therefore, when the star ratings are the main driver of bonuses to MA plans, the adjustment strategy of the ratings can’t ignore contextual factors embedded in geographic considerations (such as type of population covered and regular and close access to healthcare facilities) and reward those plans that offer tailored care to their specific geographic needs.

In a pioneering study by Wennberg & Gittelsohn [3], small area variation was demonstrated as a fundamental variable in the analysis of processes of care. Since then, several studies have documented large geographic variation in regards to health expenditure [4–6], surgical procedures [7–9] and utilization of services [10–13]. Even though the large majority of these studies adjusted their results for factors such as demographics, comorbidities and socioeconomic status, the investigation between small geographic variation and quality of care requires more attention. Moreover, our knowledge of the variation of both small and large geographic areas in terms of patient reported outcomes across insurance arrangements is still limited, although some previous evidence offers some insights. For example, Kazis et al [14] in an earlier work demonstrated small to moderate differences across Veteran Administration hospitals nationwide using the Veterans RAND 36 Item Health Survey, a Patient Reported Outcome metric that assesses a person’s physical and mental health.

Considering the proven extent of geographic variation in Medicare utilization of services [10], policymakers need a more complete understanding of the underlying sources of this variation in terms of quality of care before formulating policies to shrink them. For that purpose, the Medicare Advantage Program (MA), adopted the star ratings system in order to rank the plans in terms of quality of care and consumer satisfaction. The rankings have important implications for re-imbursement, bonuses and expansion of plan businesses. These rankings are computed based on four different data sources: (1) the Healthcare Effectiveness Data and Information Set (HEDIS®), (2) the Consumer Assessment of Healthcare Providers and Systems (CAHPS®), (3) the Medicare Health Outcomes Survey (HOS)which includes the Veterans Rand 12-Item Health Survey (VR-12), and (4) CMS administrative data which includes information about member satisfaction and disenrollment, as well as plans’ appeals processes, audit results, and customer service [15].

The MA star ratings have been proven to vary greatly in terms of hospital characteristics and profit status. Xu et al. demonstrated that for profit and non-profit status are important predictors of the Star ratings as well as the volume of subscribers in a plan and the longevity of the plan [16]. This paper aims to investigate the association between geographic variation and the MA star ratings nationally to determine the importance of geography in predicting the ratings.

## Methods

The summary MA star rating provides an overall measure of a plan’s quality based on indicators related to process of care, health outcome, access to care, and beneficiary satisfaction (Kaiser Family Foundation 2009). One star indicates poor performance, two stars means below average, three stars is average, and four and five stars signify above-average and excellent performance respectively. At the time of the study, the star rating covered 36 different topics in 5 categories: staying healthy, managing chronic (long-term) conditions, member experience, member complaints and customer service for MA. If the contract provides medication benefits only, the star rating covers 15 topics and 4 categories: customer service, member complaints, member experience, and patient safety. If the contract provides health and medication services together then the star rating covers all of the topics listed above.

### Data

Four data sources were used in this study: 1) The 2010 MA plan quality database, which consists of star rating information at the contract level. 2) The Medicare Advantage contract enrollment database, which reflects enrollment status as of October 1^st^, 2010. 3) The 2000 census databases, which was merged to the MA plan quality database to extract the demographic information described in Table 1. 4) The U.S. county FIPs code, which is a five-digit Federal Information Processing Standard (FIPS) code that uniquely identifies counties and county equivalents in the United States, certain U.S. possessions, and certain freely associated states.

**Table 1:** Population Characteristics. N = 2872 counties.

Variable	Mean (%)	Std Dev	Median (%)
Population older than age 65	14.50	3.91	14.22
White	84.38	16.11	90.89
African American	9.38	14.80	2.18
Asian	0.87	2.08	0.35
Native Indian	1.35	5.00	0.33
Hawaiian	0.06	0.38	0.03
Other race groups	2.50	4.73	0.66
Population education level is equal or above Bachelor degree	10.00	4.79	8.79
Population under poverty level (based on 2000 census standard)	13.55	6.13	12.53
Rural population	57.35	30.28	58.07
Medicare Advantage enrollment penetration	17.20	12.12	14.35
Median household income ($)	35667.18	9001.78	34181.00

### Study Data and Methods

#### Inclusion/Exclusion Criteria

We included only those contracts offering health services (“Part C” or “Part C + Part D”) and exclude the contracts related to medication only (Part D) in our computations. Such an approach allows us to make the scores comparable within contracts. Counties in Puerto Rico, Guam and Virgin Islands were not included in our study since they are technically not states. As a result, 409 (71.1%) of 575 MA contracts were included in our final study sample, representing 86.3% of the total Medicare Advantage population (11.7 million).

Institutional Review Board approval was obtained through Boston University Medical Center. Records/information was anonymized and de-identified prior to analysis.

### Unit of analysis

The analysis of this study was based upon the MA star ratings at the contract level. We began with an analysis at the FIPS level by county and contract. The U.S county serves as our unit of analysis. A county is a geographic subdivision of a state (or federal territory), usually assigned some governmental authority. County is chosen because census information is collected at the county level and Medicare Advantage contracts locate their health plan also at the county level. In subsequent analysis we also use contracts negotiated among the MA plans as a separate unit of analysis.

### County Level Analysis

This study focuses on the variation in MA star ratings associated with geography, namely U.S. counties or FIPS. First, the level of heterogeneity of the MA star ratings across 50 states was tested. Variables reflected the make-up of each contract based on the weighted values from the counties in which they operated. For each county, a weight was assigned at the Medicare Advantage contracts level accounting for the population density (i.e., contracts with more enrollees will have larger weights since they account for more Medicare Advantage market share). Then, all contracts operated in each county were averaged to obtain the aggregated MA star rating score. The methodology used was not unlike that by Schneider et al [17].

### Statistical analysis

The data analyses focused on the county level of MA contracts and differences in their star ratings in a series of steps. First, the MA star ratings were measured with a multicomponent index scale that can take on values from 1 to 5 in increments of 0.5. Because the underlying variables that comprise this index are continuous by nature, we analyzed the star ratings as a continuous variable as in previous published analysis [16].

Initial analysis applied general linear models to obtain the unadjusted MA star ratings by state. Multivariate linear regression models were weighted by counties to determine the fraction of each county contributing to the nationwide enrollee population (Weight = number of enrollees / 10.1 million × 2873 counties). Then, in univariate analyses, we examined the data set of 409 contracts with respect to age, race, education, below poverty level or not, rural vs. urban, and MA enrollment penetration, using nominal scale, categorical variables in terms of percentages. The Median household income is reported in measures of central tendency (mean and median counts).

Separately, we determined the independent (pure) effect of geography and socio-demographics on the MA star ratings using a variance components analysis in multivariate regressions. We defined total effect as the G (pure geography effect) + S (pure socio-demographics effect) + J (joint effect). Then, we calculated total sum of squares from 3 regression models: one with the G component, one with the S component and the third with both G+ S. Lastly, the independent (pure) effect of geography and socio-demographics was calculated by subtraction. We next analyzed the data using multivariate ordinary least squares regression models for the MA star ratings adjusting for demographics and geographic variation. The principal independent variable for the adjusted star ratings are reported by state, aggregating county data at the state level. Separate analysis included a mixed model to control for clustering effects at the county level using random effects (data available per request). All analyses were conducted in a SAS 9.1 environment.

## Results

In our analysis of 409 MA contracts, there were 10.6 million beneficiaries, representing 86.3% of the total Medicare Advantage population (11.7 million). The mean star rating for all contracts was 3.33, where 5 denotes excellent performance.

Table 1 reports the descriptive characteristics by the FIPs counties nationally. We observed that 14.5% of the sample were older than 65 years of age, about 84% White and 9% African American. For education, 10% had a bachelor degree or above, about 13% were below the poverty level, 57% had a rural area of residence and enrollment penetration of the MA program averaged 17%. The mean and median household income was about $35,000.

Table 2 gives the variance components analysis results. Geography alone (G) explained 59.5% of the total variance. Demographic characteristics alone (S) explained 31.2% and when combined (J), all the covariates explained 71.7% of the total variance (p<0.0001).

**Table 2:** Explained variance by Geography and Socio-demographic factors.

Model	Variables included	Adjusted r^2^
Geography effect (G)	Contract location at the county level, contract location at the state level	0.595
Socio-Demographics effect (S)	Age, race, education, poverty level, enrollment penetration, median household income	0.312
Joint Effect (G+S)	Age, race, education, poverty level, enrollment penetration, median household income, contract location at the county level, contract location at the state level	0.717

Table 3 reports the results of the FIPS county level analysis for the current MA star rating and the adjusted star rating (when geography location and population density are factored in) at the state level. Results are rank ordered by the 50 states and highlights the “difference” between the unadjusted, unweighted star rank (current MA rating) versus the adjusted, weighted star rank (adjusted MA rating). In other words, the rank difference emphasizes a different ranking across states when geography and population density estimates are both accounted for. Positive values represent an improvement in the ranking after the adjustments. Results indicated a positive change in 20% of the states (≥ 5 point differences); for 6% of the states the adjustment did not change their ranking positions and 24% changed negatively (≥5 point differences). For example, let’s describe Michigan: the current MA star rating positioned the state in the 26^th^ place compared to the other 50 states. After the adjustment, it ranked as the fourth highest in the country, a +22 point difference change. The opposite occurred for Hawaii with a -22 point difference after adjustment. Only three states did not change ranking after the adjustments. Overall adjustments for the S effect had a small impact on the rankings compared to the G effect (results available on request).

**Table 3:** Current and enhanced State level star rankings.

U.S State	Ranking difference	Current star rank	Adjusted star rank	Current star rating	Adjusted star rating
Michigan	22	26	4	3.32	3.82
New Mexico	20	25	5	3.32	3.75
Texas	15	38	23	3.06	3.37
Washington	10	16	6	3.52	3.74
Arizona	9	42	33	2.98	3.21
Nevada	9	50	41	2.44	3.04
California	8	10	2	3.74	4.10
Florida	7	37	30	3.11	3.26
Oklahoma	7	41	34	2.98	2.21
New Jersey	6	33	27	3.15	3.30
North Carolina	4	32	28	3.22	3.30
Louisiana	4	35	31	3.13	3.26
Kansas	4	44	40	2.89	3.07
Oregon	3	13	10	3.63	3.67
Maine	2	7	9	3.79	3.68
Virginia	2	23	21	3.40	3.43
Missouri	2	27	25	3.32	3.33
Alabama	2	49	47	2.72	2.79
Connecticut	1	17	16	3.52	3.53
Maryland	1	19	18	3.46	3.52
Delaware	1	40	39	3.00	3.09
Massachusetts	0	1	1	4.16	4.27
Colorado	0	8	8	3.76	3.69
Iowa	0	20	20	3.46	3.45
DC	-1	2	3	3.93	4.08
Minnesota	-1	6	7	3.84	3.70
Rhode Island	-1	11	12	3.70	3.61
North Dakota	-1	12	13	3.65	3.60
West Virginia	-1	45	46	2.82	2.80
Arkansas	-1	48	49	2.72	2.74
New York	-2	9	11	3.75	3.65
Wyoming	-2	15	17	3.52	3.53
South Carolina	-2	43	45	2.96	2.89
Mississippi	-2	46	48	2.81	2.74
Tennessee	-3	21	24	3.42	3.35
Kentucky	-3	47	50	2.74	2.74
New Hampshire	-4	18	22	3.49	3.37
Indiana	-4	39	43	3.01	3.02
South Dakota	-5	14	19	3.59	3.46
Illinois	-6	29	35	3.28	3.20
Nebraska	-7	22	29	3.41	3.27
Montana	-7	30	37	3.28	3.15
Georgia	-7	31	38	3.25	3.13
Vermont	-8	24	32	3.36	3.23
Idaho	-8	28	36	3.29	3.16
Ohio	-8	34	42	3.14	3.02
Utah	-8	36	44	3.11	2.90
Pennsylvania	-10	5	15	3.88	3.54
Wisconsin	-11	3	14	3.89	3.60
Hawaii	-22	4	26	3.88	3.32

(1) All columns are adjusted for age, race, education, economic status (including poverty level and median household income), rural/urban, and Medicare Advantage enrollment penetration.(2) The enhanced columns are also adjusted for geographic location and population density 3) The 2 horizontal lines in the table give the tertiles of state star ratings.

**Fig 1:**
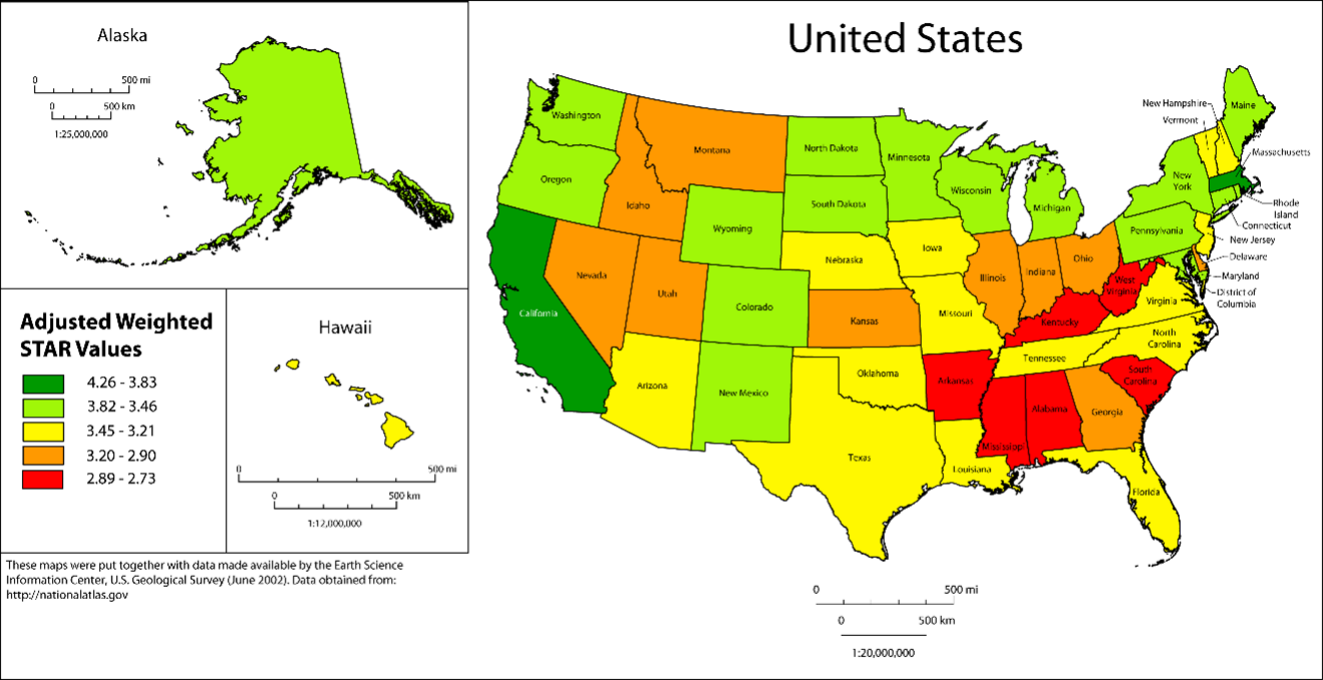
State-level star ratings scores adjusted by geography location and population density.

To assess for geographic spatial relationships, the adjusted star ratings were plotted by state (Fig 1). Results suggested lower star ratings for the southeastern and upper and lower mid sections of the U.S compared to northeastern and western states.

**Fig 2:**
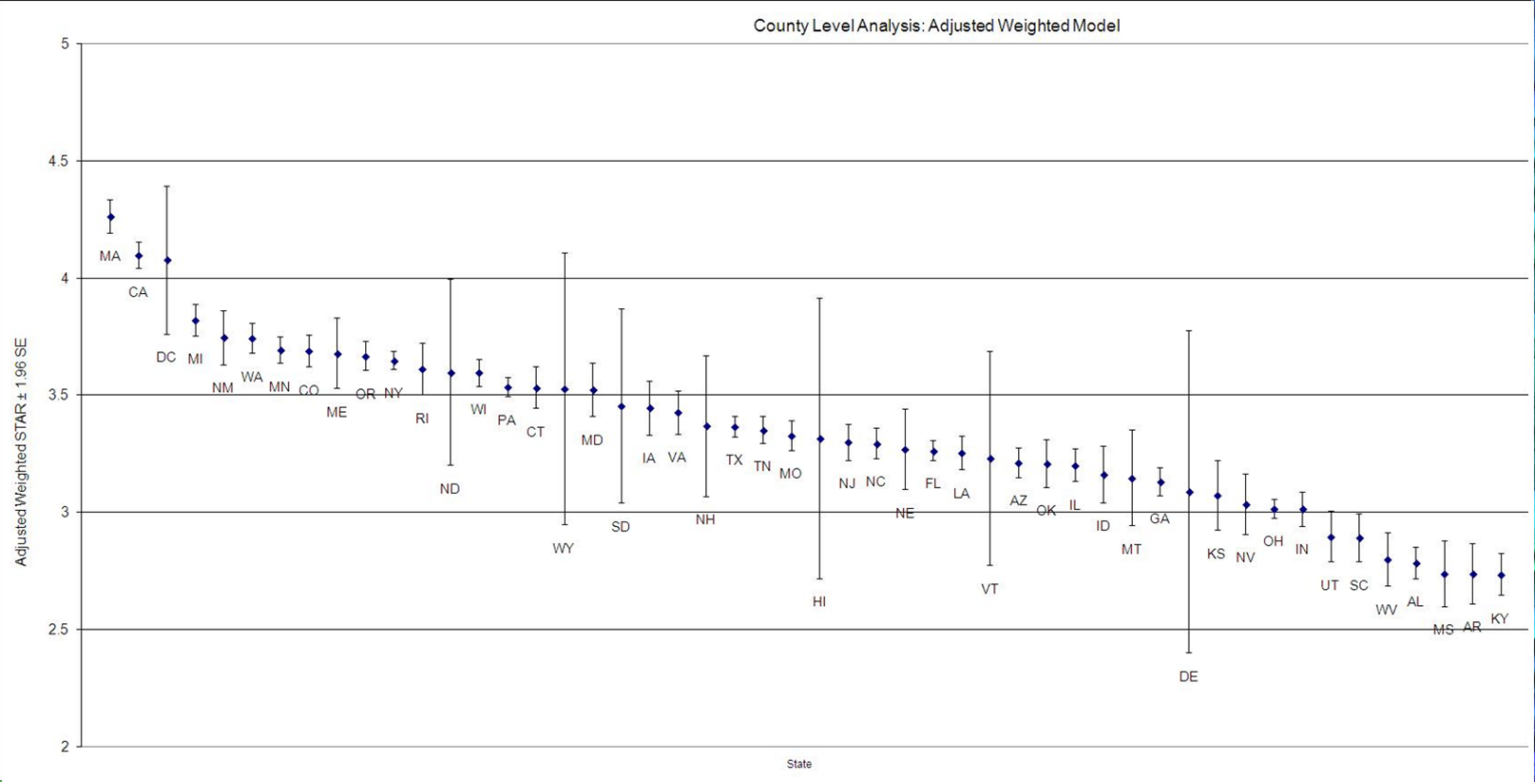
State-level star ratings scores adjusted by geography location and population density (Mean and Standard Deviation).

Finally, we reported the state-level adjusted star ratings by mean and standard deviation (Fig 2). A total of 42 states had average star ratings (between 3 and 4). Only three states ranged above-average after the adjustments. A total of seven states performed below average. When compared to current star ratings, three states went from below-average to average and one state went from average to above-average after the adjustments. In summary, the adjusted star ratings were changed positively by 0.18 and negatively by 0.15 compared to the current MA ratings.

Separately, we conducted sensitivity analyses at the contract level, to test if the contract, which can be present in more than one FIPs, may play a role on the findings about ranking variation at the state level. In total, there were 409 contracts, of these, 80 (19.6%) spanned all 50 states, 55 (13.4%) operated in a range of 2–23 states and 274 (66.9%) operated in one state only. Results from the analysis of variance in balanced data showed no significant differences between these 3 contract constellations in terms of star ratings (P = 0.32 in unadjusted models) compared with the FIPS analysis in Table 3.

## Discussion

Geographic variation in healthcare remains a pervasive, persistent and substantial concern for policymakers, providers and the public in general. This and previous work [1,4,7,12,18,19] have established that first, geographic variation is not random, second, demographics and health status reduces to some extent the variation, but much unexplained variation remains, third, contextual local factors that are geographically based need to be considered more carefully when comparisons are made among plans.

This study found extensive geographic variation throughout the U.S in terms of quality of care. Overall, almost half of the states moved ≥ 5 points above or below their original MA star rank when controlling for small area variation and demographics. Also, the geography effect explained a larger fraction of the star ratings’ variability than sociodemographic factors. Finally, these results suggest lower adjusted star ratings in mid- central and southeastern states. To our knowledge, this is the first study to aggregate the MA star ratings at the state level and assess the effects of the beneficiaries’ demographics and small area variation (FIPS counties) over the ratings.

We postulate that the geographic variability in quality of care likely arises from the interaction of several components such as the differences in the demographics and socio-economic status of populations, underlying prevalence of morbidities, differences in the approaches to treatments, and overuse and misuse of medical technologies. Thus far, the literature has provided possible explanations for these differences in quality of care, rather than systematic investigations of factors associated with geographic variability in the utilization of services and its impact on the quality of health care.

The MA star ratings are a unique opportunity to assess in a single metric relevant information for the consumer in terms of health effectiveness data, patient reported outcomes and beneficiary satisfaction. Previous work assessing geographic variation and quality have important limitations: first, many studies rely on mortality to measure performance and outcomes across geographic regions [20,21]. Mortality is reliable and valid in those conditions that have a high probability of premature death and reduction of mortality is a meaningful endpoint. However, for those conditions that are chronic and with impacts on the physical and psychological status of individuals—often seen in MA beneficiaries—increased survival in the elderly pushes research to more intermediate and long-term outcome measures with proven reliability, validity and responsiveness to change over time such as some of the components of the MA star ratings including the VR-12 physical and mental summary measures. Second, variation in quality of care should be able to distinguish between “acceptable” and “unacceptable” factors that contribute to processes of care including different comorbidity profiles among subjects, demographic characteristics and geography. Unexplained variability across geographic regions may be explained by market characteristics and discretionary provision of inefficiencies in the care process, as highlighted by the 2013 report from the Institute of Medicine [22].

It is important to recognize these factors when reaching conclusions about why utilization varies from one part of the country to another. Unfortunately, classifying the reasons behind this variation including socioeconomic status or race, are controversial adjustments because they are often seen as factors beyond the scope of the health care system, as noted by CMS. However, CMS has also been diligent in introducing changes to the MA star ratings yearly. For example, mammography will be introduced in the 2016 version of the ratings. This change reflects CMS’ ongoing efforts to reflect broader appropriateness measures [23].

In addition, this study found substantial quality of care variation across counties, which warrants future studies with deeper analysis in terms of the underlying factors driving this variability.

It remains unclear as to the degree of geographic variation that should be considered relevant from a societal or health system perspective. For geography, the amount of variability reported is different across studies with differences in health status ranging from 18% [4] to almost 70% [6]. Such variability is partly explained by the different geographical unit of analysis and the risk adjustment methodology. In addition to individual characteristics or insurance provisions, other factors such as local and regional markets for pharmaceuticals, supply of providers, hospital size, and level of competition among institutions may have an impact on quality of care. For example, Wennberg and colleagues found that higher ratios of beds per capita (larger institutions) are associated with higher health care utilization in inpatient settings [24]. Another study found that higher percentage of PCPs predict lower spending per beneficiary within a region [25].

Overall, the vast majority of studies address the regional variability in terms of price [26–28]. However the measurement of quality of care across different geographical locations is sparse in the published literature. Even though the price factor is an important predictor for MA as a system, quality measurement across regions in the country would allow for the development of more sensitive and useful indicators for the consumer, and more importantly, identify avoidable utilization, which may be easier to address than price [18].

Practice of medicine may also help explain the geographic variation. Proposed guidelines and formularies may raise controversies within provider’s practices about alternative therapies and additional information may be needed to settle these differences. Describing geographic variation does not necessarily provide solutions. Also, different concepts may be considered “proper treatment” in different regions. For example, provider groups located in urban areas may be more willing to adapt to more innovative and newer treatment options compared to the willingness of providers practicing medicine in more rural, isolated areas. Therefore, more studies are needed at finer levels of granularity. For example, Barnato et al [29] explored treatment choices for critically ill patients in Pittsburg and found clinicians overestimating the patient’s preference for intensive treatment. These type of preferences may be contextual of clinicians in Pittsburg and may not hold true in other regions. Thus, finding “hot spots” of overuse is a good example of policy relevant interventions that can be planned when small geographic variation is identified, improving the likelihood of fairer comparisons.

The characteristics of the insurance arrangement within MA may also have a role in the geographic variation. Even though previous studies did not find an association between plan characteristics and quality metrics [30], no previous literature explored the interactions between regions and insurance arrangements, which might shed light about different region/insurance mechanisms. A report about commercial insurance in the U.S related to depression treatment found that HMOs in the South were less likely to offer guideline concordant care compared to HMOs in the West unpublished data). Hence, more research is needed to disentangle the contextual factors impacting on the insurance plans’ operations.

One of the main aspects of the debate in 2009 around the passing of the Affordable Care Act is the relationship between geographic variation and healthcare cost[19]. One of the main objectives of the law was universal coverage of health insurance, however concerns were raised over the additional costs of this coverage. The discussion included identifying geographic variation in terms of costs and health outcomes (mortality) to reduce over-use of supply-sensitive services and transform high cost, ineffective regions to low-cost, highly efficient care with quality services [19]. Mangione and colleagues [31] rather than using only mortality to assess variation of care across 6 hospitals, decided to measure survival after heart failure finding higher survivability rates among teaching hospitals with increased resource use. They concluded, “much more work is needed to truly distinguish inefficient from beneficial resource use” [31].

We should acknowledge several limitations for this study. First, our findings may not be generalize to health care systems with different structure, financial incentives, or provider training than MA. Therefore, our findings may not generalize to health contracts covering other populations, such as Medicaid and commercial enrollees. Second, our sample was mostly represented by those MA enrollees 75 years or younger, as a result the findings of this study may not be generalizable to those above 75 years of age. Several measurements in the Medicare plan star ratings were revised in 2012. As the CMS continues to provide updates on the ratings each year, we do not expect that star ratings changes would alter our major results in important ways. Third, our results would be strengthened if the CMS referenced the data available with various data components that are aggregated into the star ratings. However, the 2010 individual component or domain data were not publicly available at the time this study was done. Future studies that examine how contract characteristics may affect specific individual measures will be of great clinical value. Although we realize that such studies should also include the point of view of both patients and medical providers, we focused on star ratings in this study.

The evaluation of quality at the aggregated level allows a better “prioritization” of resources when comparisons are made at the county or state level. The results of this work highlight the need of contemplating the introduction of better tools to quantify and explore what drives this substantial geographic variation.

## Conclusion

The current health reform underway in the United States focuses on changing the behavior of individual providers to increase access to health services, improve quality of care and decrease healthcare costs. The results of this paper suggest that the rationale for a geographical focus is strong, to the extent that the explained variation detected may reflect a number of factors some of which may include system inefficiencies, local context or diverse health care processes. The factors associated with population health in different geographic areas are largely local, rooted in the environmental, social, economic, and behavioral determinants of health. These proposed changes should encourage CMS to consider stratification by geographic units to allow fairer comparisons among plans. The pursuit of better quality of care should incentivize the use of more refined approaches such as use of geospatial statistical methods allowing the introduction of local information in the calculation of composite measures such as the star ratings.

## References

[1] Fisher ES, Wennberg JE. Health care quality, geographic variations, and the challenge of supply-sensitive care. Perspect Biol Med. 2003;46: 69–79. 12582271 10.1353/pbm.2003.0004

[2] Atkinson R, Kintrea K. Disentangling Area Effects: Evidence from Deprived and Non-deprived Neighbourhoods. Urban Stud Routledge. 2001;38: 2277–2298. 10.1080/00420980120087162

[3] Wennberg J, Gittelsohn A. Small Area Variations in Health Care Delivery A population-based health information system can guide planning and regulatory decision-making. Science. 1973;182: 1102–1108. 4750608 10.1126/science.182.4117.1102

[4] Congress of The United States, Congressional Budget Office. Geographic Variation in Health Care Spending [Internet]. 2008 Feb. Available: http://www.cbo.gov/sites/default/files/cbofiles/ftpdocs/89xx/doc8972/02-15-geoghealth.pdf

[5] Fisher ES, Bynum JP, Skinner JS. Slowing the growth of health care costs—lessons from regional variation. N Engl J Med. 2009;360: 849–852. 10.1056/NEJMp0809794 19246356 PMC2722744

[6] Reschovsky JD, Hadley J, O’Malley AJ, Landon BE. Geographic Variations in the Cost of Treating Condition-Specific Episodes of Care among Medicare Patients. Health Serv Res. 2014;49: 32–51. 10.1111/1475-6773.12087 23829388 PMC3922465

[7] Daffner SD, Beimesch CF, Wang JC. Geographic and demographic variability of cost and surgical treatment of idiopathic scoliosis. Spine. 2010;35: 1165–1169. 10.1097/BRS.0b013e3181d88e78 20421853

[8] Goodney PP, Travis LL, Malenka D, Bronner KK, Lucas FL, Cronenwett JL, et al Regional variation in carotid artery stenting and endarterectomy in the Medicare population. Circ Cardiovasc Qual Outcomes. 2010;3: 15–24. 10.1161/CIRCOUTCOMES.109.864736 20123667 PMC5240818

[9] Miller DC, Gust C, Dimick JB, Birkmeyer N, Skinner J, Birkmeyer JD. Large variations in Medicare payments for surgery highlight savings potential from bundled payment programs. Health Aff (Millwood). 2011;30: 2107–2115. 22068403 10.1377/hlthaff.2011.0783 PMC4003905

[10] Parker L, Levin DC, Frangos A, Rao VM. Geographic variation in the utilization of noninvasive diagnostic imaging: national Medicare data, 1998–2007. Am J Roentgenol. 2010;194: 1034–1039. 20308507 10.2214/AJR.09.3528

[11] Curtis LH, Greiner MA, Patel MR, Duncan PW, Schulman KA, Matchar DB. Geographic variation and trends in carotid imaging among Medicare beneficiaries, 2001 to 2006. Circ Cardiovasc Qual Outcomes. 2010; CIRCOUTCOMES–110. 10.1161/CIRCOUTCOMES.110.950279 20940248

[12] Ashton CM, Petersen NJ, Souchek J, Menke TJ, Yu HJ, Pietz K, et al Geographic variations in utilization rates in Veterans Affairs hospitals and clinics. N Engl J Med. 1999;340: 32–39. 9878643 10.1056/NEJM199901073400106

[13] Burke JF, Kerber KA, Iwashyna TJ, Morgenstern LB. Wide variation and rising utilization of stroke magnetic resonance imaging: data from 11 states. Ann Neurol. 2012;71: 179–185. 10.1002/ana.22698 22367989 PMC3297973

[14] Kazis LE, Miller DR, Skinner KM, Lee A, Ren XS, Clark JA, et al Patient-reported measures of health: the Veterans Health Study. J Ambulatory Care Manage. 2004;27: 70–83. 14717468 10.1097/00004479-200401000-00012

[15] Kazis LE, Selim AJ, Rogers W, Qian SX, Brazier J. Monitoring outcomes for the Medicare Advantage Program: Methods and application of the VR-12 for evaluation of plans. J Ambulatory Care Manage. 2012;35: 264–277. 10.1097/JAC.0b013e318267468f 22955087

[16] Xu P, Burgess J, James F., Cabral H, Soria-Saucedo R, Kazis LE. Relationships Between Medicare Advantage Contract Characteristics and Quality-of-Care RatingsAn Observational Analysis of Medicare Advantage Star RatingsContract Characteristics and the Delivery of Quality of Care. Ann Intern Med. 2015;162: 353–358. 10.7326/M14-0332 25732277

[17] Schneider EC, Zaslavsky AM, Epstein AM. Quality of care in for-profit and not-for-profit health plans enrolling Medicare beneficiaries. Am J Med. 2005;118: 1392–1400. 10.1016/j.amjmed.2005.05.032 16378784

[18] Institute of Medicine. Making Sense Of Geographic Variations In Health Care: The New IOM Report. In: Health Affairs Blog [Internet]. 2013 [cited 15 Jun 2014]. Available: http://healthaffairs.org/blog/2013/07/24/making-sense-of-geographic-variations-in-health-care-the-new-iom-report/

[19] Rosenthal T. Geographic Variation in Health Care. Annu Rev Med. 2012;63: 493–509. 10.1146/annurev-med-050710-134438 22053738

[20] Allen NB, Holford TR, Bracken MB, Goldstein LB, Howard G, Wang Y, et al Geographic Variation in One-Year Recurrent Ischemic Stroke Rates for Elderly Medicare Beneficiaries in the USA. Neuroepidemiology. 2010;34: 123–129. 10.1159/000274804 20068358 PMC2837886

[21] Welch H, Sharp SM, Gottlieb DJ, Skinner JS, Wennberg JE. GEographic variation in diagnosis frequency and risk of death among medicare beneficiaries. JAMA. 2011;305: 1113–1118. 10.1001/jama.2011.307 21406648 PMC3071496

[22] Institute of Medicine of the National Academies. Variation in Health Care Spending: Target Decision Making, Not Geography [Internet]. 2013. Available: http://www.iom.edu/Reports/2013/Variation-in-Health-Care-Spending-Target-Decision-Making-Not-Geography.aspx 24851301

[23] Centers for Medicare & Medicaid Services. Enhancements to the Star Ratings for 2016 and Beyond [Internet]. enters for Medicare & Medicaid Services; 2014 Nov. Available: http://www.cms.gov/Medicare/Prescription-Drug-Coverage/PrescriptionDrugCovGenIn/Downloads/2016-Request-for-Comments-v-11_25_2014.pdf

[24] Welch WP, Miller ME, Welch HG, Fisher ES, Wennberg JE. Geographic variation in expenditures for physicians’ services in the United States. N Engl J Med. 1993;328: 621–627. 10.1056/NEJM199303043280906 8429854

[25] Baicker K, Chandra A. Medicare Spending, The Physician Workforce, And Beneficiaries’ Quality Of Care. Health Aff (Millwood). 2004; 10.1377/hlthaff.w4.184 15451981

[26] Morden NE, Schwartz LM, Fisher ES, Woloshin S. Accountable Prescribing. N Engl J Med. 2013;369: 299–302. 10.1056/NEJMp1301805 23883375 PMC3855284

[27] Likosky DS, Zhou W, Malenka DJ, Borden WB, Nallamothu BK, Skinner JS. Growth in medicare expenditures for patients with acute myocardial infarction: A comparison of 1998 through 1999 and 2008. JAMA Intern Med. 2013;173: 2055–2061. 10.1001/jamainternmed.2013.10789 24061277 PMC4454469

[28] Brown JR, Sox HC, Goodman DC. Financial incentives to improve quality: Skating to the puck or avoiding the penalty box? JAMA. 2014;311: 1009–1010. 10.1001/jama.2014.421 24618957 PMC6628700

[29] Barnato AE, Mohan D, Downs J, Bryce CL, Angus DC, Arnold RM. A randomized trial of the effect of patient race on physiciansʼ intensive care unit and life-sustaining treatment decisions for an acutely unstable elder with end-stage cancer*: Crit Care Med. 2011;39: 1663–1669. 10.1097/CCM.0b013e3182186e98 21460710 PMC3119368

[30] Qato DM, Trivedi AN. Receipt of high risk medications among elderly enrollees in Medicare Advantage plans. J Gen Intern Med. 2013;28: 546–553. 10.1007/s11606-012-2244-9 23129159 PMC3599014

[31] Ong MK, Mangione CM, Romano PS, Zhou Q, Auerbach AD, Chun A, et al Looking Forward, Looking Back Assessing Variations in Hospital Resource Use and Outcomes for Elderly Patients With Heart Failure. Circ Cardiovasc Qual Outcomes. 2009;2: 548–557. 10.1161/CIRCOUTCOMES.108.825612 20031892 PMC2951887

